# Efficacy of fluid loading as a countermeasure to the hemodynamic and hormonal changes of 28‐h head‐down bed rest

**DOI:** 10.14814/phy2.13874

**Published:** 2018-10-08

**Authors:** Heather Edgell, Anna Grinberg, Keith R. Beavers, Nathalie Gagné, Richard L. Hughson

**Affiliations:** ^1^ Faculty of Applied Health Sciences University of Waterloo Waterloo Ontario Canada; ^2^ School of Kinesiology and Health Sciences York University Toronto Ontario Canada; ^3^ Schlegel‐University of Waterloo Research Institute for Aging Waterloo Ontario Canada

**Keywords:** Aldosterone, brachial blood flow, lower body negative pressure, orthostatic stress, plasma volume, renin

## Abstract

After exposure to microgravity, or head‐down bed rest (HDBR), fluid loading is often used with the intent of increasing plasma volume and maintaining mean arterial pressure during orthostatic stress. Nine men (aged 18–32 years) underwent three randomized trials with lower body negative pressure (LBNP) before and after: (1) 4‐h of sitting with fluid loading (1 g sodium chloride/125 mL of water starting 2.5‐h before LBNP), (2) 28‐h of 6‐degree HDBR without fluid loading, and (3) 28‐h of 6‐degree HDBR with fluid loading. LBNP was progressive from 0 to −40 mmHg. After 28‐h HDBR, fluid loading did not protect against the loss of plasma volume (−280 ± 64 mL without fluid loading, −207 ± 86 with fluid loading, *P* = 0.472) nor did it protect against a drop of mean arterial pressure (*P* = 0.017) during LBNP (Post‐28 h HDBR response from 0 to −40 mmHg LBNP: 88 ± 4 to 85 ± 4 mmHg without fluid loading and 93 ± 4 to 88 ± 5 mmHg with fluid loading, *P* = 0.557 between trials). However, fluid loading did protect against the loss of stroke volume index and central venous pressure observed after 28‐h HDBR. Fluid loading also attenuated the increase of angiotensin II seen after 28‐h HDBR and throughout the LBNP protocol (Post‐28 h HDBR response from 0 to −40 mmHg LBNP: 16.6 ± 3.4 to 23.7 ± 5.0 pg/mL without fluid loading and 6.1 ± 0.8 to 12.2 ± 2.3 pg/mL with fluid loading, *P* < 0.001 between trials). Our results indicate that fluid loading did not protect against plasma volume loss due to HDBR or change blood pressure responses to LBNP. However, changes in central venous pressure, stroke volume and fluid regulatory hormones could potentially influence longer duration studies and those with more severe orthostatic stress.

## Introduction

After exposure to microgravity, astronauts experience many physiological changes such as reductions in skeletal muscle mass (Narici et al. 2003) and bone mineral density (Orwoll et al. 2013) alongside orthostatic hypotension (i.e., fainting) upon return to Earth due to cardiovascular deconditioning (Buckey et al. 1996). Astronauts returning to Earth who did not faint upon an orthostatic challenge were able to maintain mean arterial pressure by adequately increasing peripheral resistance (Buckey et al. 1996; Fritsch‐Yelle et al. 1996; Waters et al. 2002; Meck et al. 2004) indicating that changes in plasma volume and/or dysregulation of arterial vasoconstriction and vasoactive hormones could play a role in orthostatic hypotension. Exposure to either acute or prolonged microgravity leads to greater cardiac output and stroke volume (Petersen et al. 2011; Hughson et al. 2017) due to cephalic fluid shifts and changes in transmural pressure gradients. Fluid shifts lead to a decrease of plasma volume in as little as 4‐h (using head‐down bed rest (HDBR); a commonly used Earth‐based model of microgravity) (Edgell et al. 2012) leading to further changes in peripheral resistance, regional blood flow, and vasoactive plasma hormone concentrations (Harrison et al. 1985; Butler et al. 1990; Frey et al. 1994; Vernikos and Convertino 1994; Hargens and Watenpaugh 1996; Greenleaf et al. 2000; Iwasaki et al. 2000; Millet et al. 2001; Perhonen et al. 2001; Arbeille et al. 2005; Bleeker et al. 2005; Waters et al. 2005; Edgell et al. 2007, 2012; Fischer et al. 2007; Hughson et al. 2016). Further, it has been hypothesized that the central venous pressure operating point is reset to a lower level after exposure to microgravity (Convertino et al. 2001) which could help to explain the inability to maintain blood pressure via diminished venous return. Indeed, participants exposed to either HDBR or spaceflight for ≥4 days have lower plasma volume with greater activation of the renin‐angiotensin‐aldosterone system (RAAS) to maintain central volume at this lower operating point (Norsk et al. 1995; Millet et al. 2001; Hughson et al. 2016).

It has been speculated that the restoration of plasma volume could restore vasoconstrictor reserve and therefore restore the ability to increase peripheral resistance during orthostatic stress and maintain hemodynamics to prevent orthostatic hypotension (Convertino et al. 2006). In 1977, Hyatt and West found that consumption of oral saline (beef bouillon) increased plasma volume after 7 days of HDBR (Hyatt and West 1977). Subsequently, the standard NASA fluid loading protocol (i.e., oral consumption of 1 g salt tablet per 125 mL water with a total volume of 15 mL/kg within a 2‐h period prior to orthostatic challenge) was found to restore plasma volume and hemodynamics after 12 days of HDBR (Waters et al. 2005); however, Waters et al. (2005) did not investigate a HDBR control group without fluid loading, nor did they control for inactivity or circadian rhythm. Convertino et al. (2001) found that rhesus monkeys exposed to head‐down tilt for 48‐h had lower central venous pressure than control animals, and that 2 h of an isotonic saline infusion increased central venous pressure to a similar magnitude in both groups despite the lower pressure in tilted animals. These findings suggest that fluid loading would be ineffective in preserving plasma volume or central venous pressure after exposure to microgravity in humans.

We hypothesized that (1) 28‐h of HDBR would result in a loss of plasma volume and therefore decreased stroke volume and central venous pressure with increased heart rate in response to an orthostatic challenge, and (2) the NASA fluid loading protocol would be ineffective against plasma volume loss and these hemodynamic changes during HDBR due to compensatory natriuretic/diuretic mechanisms and resetting of the central venous pressure operating point.

## Materials and Methods

### Ethical approval and participant description

Nine healthy men (74 ± 2 kg, 176 ± 2 cm) between the ages of 18 and 32 years participated in this study that was approved by the Office of Research Ethics at the University of Waterloo. Informed consent and general health questionnaires were obtained prior to the first testing day. All participants were free from previously diagnosed cardiovascular disease and refrained from caffeine, alcohol, heavy exercise, and medication use for 24 h prior to testing.

### Bed rest/seated protocols

All trials began with measurements of plasma volume and a lower body negative pressure (LBNP) test (described below) followed by one of three randomized trials: (1) 4 h sitting with fluid loading (FL‐SEAT) to account for circadian and/or inactivity effects, (2) two different 28 h HDBR trials (6°) were conducted with (FL‐HDBR) or without fluid loading (NFL‐HDBR). Pretrial LBNP testing (“Control”) was performed at ~9:00 am and posttrial LBNP was performed at ~1:00 pm. The HDBR and seated trials were completed in random order with a minimum of 48 h between test dates. All nine men completed every trial.

Subjects consumed 5 mL/kg of water the night prior to testing and the morning before each trial to maintain hydration. Testing for all trials began at 7:00 am, 1 h after eating a light breakfast which was kept consistent between testing days. During the 28‐h trials, participants also drank 5 mL/kg of water before sleep at 11:00 pm and after waking up at 6:30 am. Water intake was ad libitum from hour 0–1.5 for all trials, and from hour 24–25.5 for the HDBR trials. Other than the aforementioned fluid loads, no water was consumed from hour 1.5–4 (all trials) or from hour 25.5–28 (HDBR trials). Diet was kept consistent between HDBR trials (2500 calories with 4 g sodium), and the lunch provided was the same for all trials. Water intake was also ad libitum from hour 4–24 on the HDBR testing days, and the second HDBR trial was matched to the first HDBR trial to within 25%.

### Lower body negative pressure protocol

Participants underwent lower body negative pressure (LBNP) as a model for orthostatic stress before and after sitting or bed rest. The participant lay supine in the LBNP chamber to the level of the iliac crest and the chamber was sealed using a neoprene kayak skirt and a belt. The LBNP protocol consisted of a 5‐min baseline period followed by 3 min of −10 mmHg, 5 min of −20 mmHg, 3 min of −30 mmHg, and 5 min of −40 mmHg. The LBNP was stopped if systolic pressure dropped to <70 mmHg, heart rate dropped by >25 bpm, or if the participant experienced nausea, dizziness, or light‐headedness. Blood samples were taken from 3.5 to 5 min of 0 mmHg, −20 mmHg, and −40 mmHg, thus necessitating the longer times at these levels of LBNP.

### Fluid loading protocol

The NASA fluid loading (FL) protocol was followed (Waters et al. 2005). This involves the ingestion of 15 mL/kg of water taken with sodium chloride capsules equivalent to 1 g/125 mL of water in the period of 2.5–0.5‐h prior to the posttrial LBNP. Salt and water were administered in equal intervals across the fluid loading period.

### Blood volume measurements

A 20‐gauge catheter (BD Insyte, BD Medical Systems, Sandy, UT, USA) was inserted into the antecubital vein of the right arm. Blood volume was determined in the seated position using the previously described carbon monoxide rebreathing technique (Burge and Skinner (1995) and used in this laboratory (Fischer et al. 2007; Edgell et al. 2012)). Changes in blood/plasma volume throughout the SEAT or HDBR protocols were determined using changes in hematocrit.

### Cardiovascular measurements

All signals were obtained using a PowerLab and LabChart software (ADInstruments, Colorado Springs, CO, USA). Beat‐to‐beat blood pressure was obtained using finger‐cuff plethysmography (Finometer; Finapres Medical Systems, Arnheim, The Netherlands) and was calibrated to a manual measurement for each LBNP session. Heart rate was determined from the R‐R interval of a single lead electrocardiogram (Pilot 9200, Colin Medical Instruments Corp., San Antonio, TX, USA). Blood flow velocity from the aortic root and the brachial artery were measured using 2 and 4 MHz Doppler ultrasound probes, respectively (Model 500M, Multigon Industries Inc., Yonkers, NY, USA). Stroke volume was determined by multiplying the cross‐sectional area and the blood flow velocity of the aorta. Stroke volume was normalized to body surface area to calculate stroke volume index. Cardiac output index was determined as stroke volume index multiplied by heart rate. Total peripheral resistance index was calculated as mean arterial pressure divided by cardiac output index. Brachial flow was determined from the cross‐sectional area and the blood flow velocity of the brachial artery. One minute averages were obtained for presentation.

### Central venous pressure

Central venous pressure was measured throughout the LBNP test using the dependent arm technique (Gauer and Sieker 1956) where individuals were supine in the LBNP box and the entire box was then tilted to the right. The antecubital catheter which was earlier used for blood volume measurements was connected to a saline filled line and a pressure transducer (TranStar, Medex Inc., Carlsbad, CA, USA). The pressure transducer was maintained at the level of the right atrium using a laser level on a tripod. Central venous pressure was calibrated to cmH_2_O using the laser level and was converted to mmHg using 1cmH_2_O=0.7355 mmHg.

### Imaging

Cross‐sectional areas of the brachial artery and aortic root were obtained using B‐mode ultrasound imaging with a HFL38 transducer (6–13 MHz) and a P17 transducer (1–5 MHz), respectively (Micromaxx, Sonosite Inc., Bothell, WA, USA). All images were captured to video tape (Sony Handycam, Model DCR‐HC42) and subsequently digitized (Adobe Premier 6.5; Adobe Systems Inc, San Jose, CA, USA). Diameters were measured using pixel coordinates of the proximal and distal vessel walls (Microsoft Paint 5.1; Microsoft Corporation, Mississauga, ON, Canada).

### Plasma hormones measurements

Blood was obtained as previously described (Edgell et al. 2012) for measurement of plasma hormones during the LBNP testing. Samples were collected, added to anticoagulant, and then centrifuged at 3000 *g* for 10 min at room temperature to obtain plasma (angiotensin II: 25 *μ*L/mL EDTA with 20 *μ*L/mL bestatin; vasopressin, renin: 25 *μ*L/mL EDTA; atrial natriuretic peptide: 37.5 *μ*L/mL EDTA and 200 KIU aprotinin/mL; norepinephrine: 25 mL/EDTA with glutathione). Plasma renin was measured by radioimmunoassay (Active Renin IRMA, Diagnostic Systems Laboratories, Inc., Webster, TX, USA), vasopressin was measured by radioimmunoassay (Vasopressin Direct RIA, ALPCO Diagnostics, Windham, NH, USA), angiotensin II was measured by enzyme immunoassay (Angiotensin II Enzyme Immunoassay Kit, Société de Pharmacologie et d'Immunologie – BIO, Montigny Le Bretonnoux, France), atrial natriuretic peptide was measured by radioimmunoassay (ALPCO Diagnostics, Windham, NH, USA), and norepinephrine was measured by high performance liquid chromatography. Aldosterone was measured in serum by radioimmunoassay (Coat‐a‐count aldosterone RIA Kit, Diagnostic Products Corporation, Los Angeles, CA, USA).

### Sodium and osmolality

Serum sodium was measured with a sodium micro probe (Orion 9811BN, Thermo Fisher Scientific, Inc., Waltham, MA, USA), and osmolality was measured by vapor pressure osmometry (Model 5100C, Wescor Inc., Logan, UT, USA).

### Design and statistical analysis

The current study was designed to examine the effects of a fluid loading protocol on the consequences of 28‐h of head‐down bed rest (HDBR). There were three pretrial LBNP tests performed. All pretrial tests were compared using one or two‐way repeated measures ANOVAs as appropriate. The only variable showing a difference after comparing pretrial responses was stroke volume index where FL‐SEAT was significantly greater than NFL‐HDBR at −30 and −40 LBNP (*P* < 0.03). The three pretrial tests were averaged together for presentation in tables and figures as “Control”.

For plasma volume and serum analysis, we compared protocols using one‐way repeated measures ANOVAs (subject as the repeated measure) followed by Tukey post hoc tests as appropriate. For hemodynamic analysis, we compared protocols using two‐way repeated measures ANOVAs (subject and protocol as repeated measures) followed by Tukey post hoc tests as appropriate. Analysis was completed using Sigmaplot 13.0 analysis software (Cary, USA). *P*‐values less than 0.05 are indicated as statistically significant. Data are presented as mean ± SE. To simplify the figures, statistical effects of LBNP are not shown.

## Results

Both 28‐h HDBR trials resulted in lower plasma volume compared to the 4‐h seated position (*P* ≤ 0.005); however, the reduction of plasma volume due to 28 h HDBR was not different with fluid loading (*P* = 0.472). There was no difference between all trials for change in serum osmolality (*P* = 0.366) or change in serum sodium concentration (*P* = 0.143; Table 1).

**Table 1:** Change in plasma volume, serum osmolality, and serum [Na+]

Trial	NFL‐HDBR	FL‐HDBR	FL‐SEAT
Plasma volume (mL)	−280 ± 64*	−207 ± 86*	+152 ± 39
Serum osmolality (mOsm)	−3.5 ± 1.5	−0.8 ± 0.9	−2.1 ± 1.3
Serum Na+ (mEq)	−0.2 ± 0.6	+2.4 ± 0.9	+0.1 ± 1.2

NFL‐HDBR is nonfluid loading after head‐down bed rest; FL‐HDBR is fluid loading after head‐down bed rest; FL‐SEAT is fluid loading after sitting; *indicates a difference compared to FL‐SEAT.

**Figure 1:**
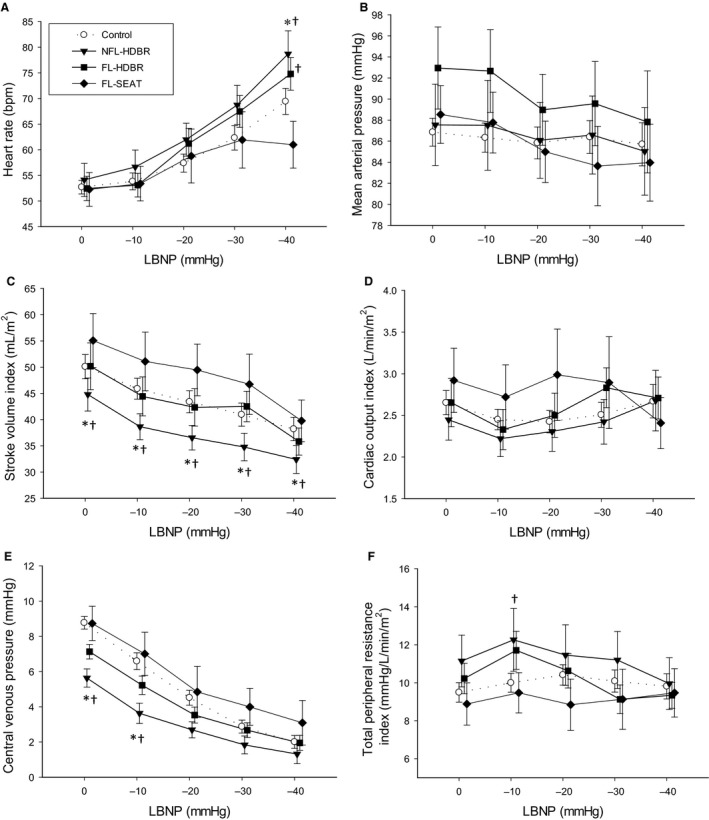
Effects of 28 h head‐down bed rest (HDBR) on heart rate (A), mean arterial pressure (B), stroke volume index (C), cardiac output index (D), central venous pressure (E) and total peripheral resistance index (F) responses to lower body negative pressure (LBNP). Symbols: Control trial (white circles with dashed lines), Nonfluid loading HDBR (NFL‐HDBR, black triangles), Fluid loading HDBR (FL‐HDBR, black squares), and Fluid loading seated (FL‐SEAT, black diamonds). *indicates a significant difference from Control; †indicates a significant difference from FL‐SEAT.

**Figure 2:**
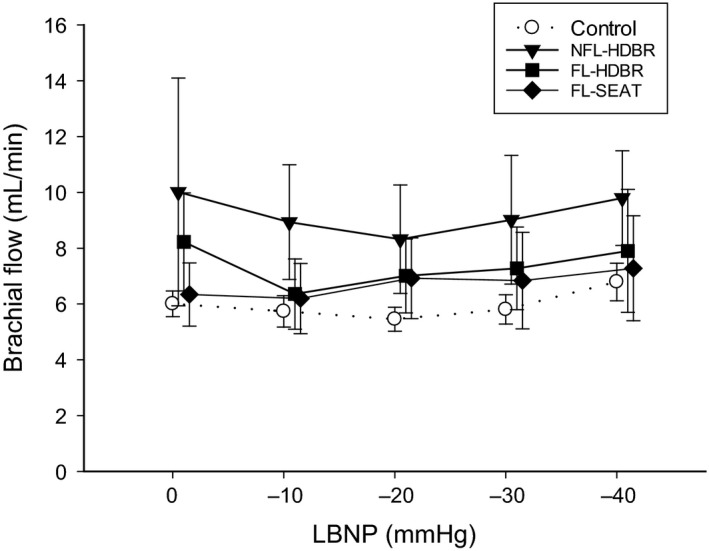
Effect of 28‐h head‐down bed rest (HDBR) on brachial blood flow responses to lower body negative pressure (LBNP). Symbols: Control trial (white circles with dashed lines), Nonfluid loading HDBR (NFL‐HDBR, black triangles), Fluid loading HDBR (FL‐HDBR, black squares), and Fluid loading seated (FL‐SEAT, black diamonds).

Heart rate increased during LBNP in all trials (*P* < 0.001, 0 vs. −40 mmHg LBNP). In both HDBR trials, participants had higher heart rate than the FL‐SEAT trial at −40 mmHg (*P* ≤ 0.01), and the NFL‐HDBR trial had higher heart rate than Control at −40 mmHg (*P* = 0.005; Fig. 1A). Mean arterial pressure decreased during LBNP in all trials (*P* = 0.017, 0 vs. −40 mmHg LBNP) with no differences between trials (*P* = 0.557, Fig. 1B). Stroke volume index decreased during LBNP in all trials (*P* < 0.001, 0 vs. −40 mmHg LBNP). The NFL‐HDBR trial had lower stroke volume index at all time points compared to Control (*P* = 0.037) and FL‐SEAT trials (*P* < 0.001; Fig. 1C). There was no effect of LBNP on cardiac output index in any trial (*P* = 0.102) nor were there any differences between trials (*P* = 0.278; Fig. 1D). Central venous pressure decreased during LBNP in all trials (*P* < 0.001, 0 vs. −40 mmHg LBNP). At 0 and −10 mmHg of LBNP, the NFL‐HDBR trial had lower central venous pressure compared to the Control and FL‐SEAT trials (*P* ≤ 0.01; Fig. 1E). There was no effect of LBNP on total peripheral resistance in all trials (*P* > 0.05; 0 vs. −40 mmHg). At −10 mmHg, the NFL‐HDBR trial had higher total peripheral resistance index than the FL‐SEAT trial (*P* = 0.026; Fig. 1F). There was no effect of LBNP on brachial flow in all trials (*P* = 0.152) nor were there any differences between trials (*P* = 0.442; Fig. 2).

**Figure 3:**
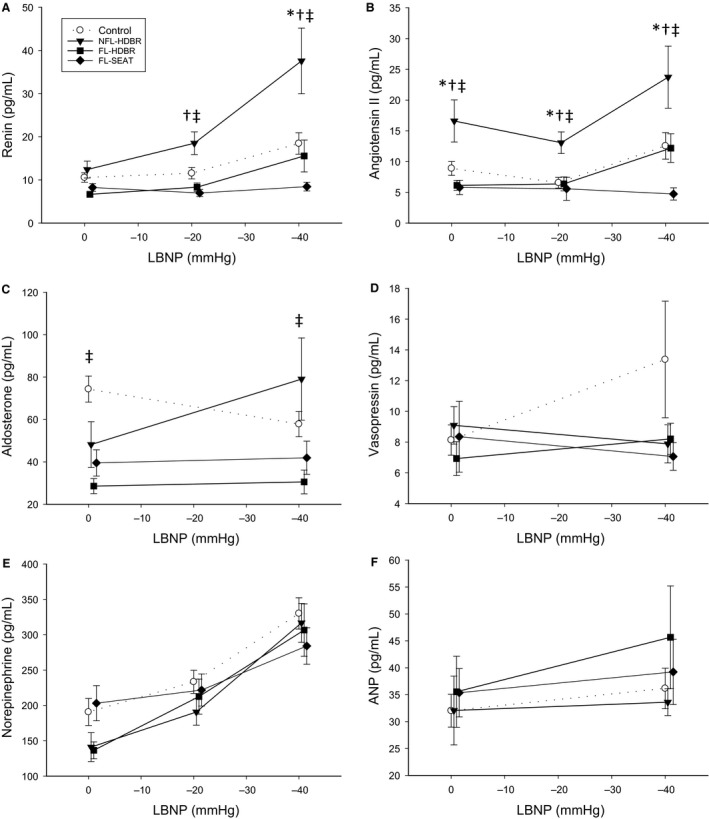
Effect of 28‐h head‐down bed rest (HDBR) on renin (A), angiotensin II (B), aldosterone (C), vasopressin (D), norepinephrine (E) and atrial natriuretic peptide (ANP; F) responses to lower body negative pressure (LBNP). Symbols: Control trial (white circles with dashed lines), Nonfluid loading HDBR (NFL‐HDBR, black triangles), Fluid loading HDBR (FL‐HDBR, black squares), and Fluid loading seated (FL‐SEAT, black diamonds). *indicates a significant difference from Control at that time point; ^†^indicates a significant difference from FL‐SEAT at that time point; ^‡^indicates a significant difference from FL‐HDBR at that time point.

There was a significant interaction between LBNP and trial for the renin response. Renin in the NFL‐HDBR trial was elevated at −20 mmHg LBNP compared to the FL‐SEAT and FL‐HDBR trials (*P* = 0.014 and *P* = 0.022, respectively) and compared to the Control, FL‐SEAT, and FL‐HDBR trials at −40 mmHg LBNP (*P* < 0.001 for all). There was an increase of renin due to −40 mmHg of LBNP in the Control (*P* = 0.044), NFL‐HDBR (*P* < 0.001), and FL‐HDBR (*P* = 0.030) trials; however, there was no increase in the FL‐SEAT trial (*P* = 0.999; Fig. 3A). At all time points, angiotensin II was elevated in the NFL‐HDBR trial compared to Control (*P* = 0.005), FL‐HDBR (*P* < 0.001), and FL‐SEAT (*P* < 0.001). Angiotensin II was higher at −40 mmHg compared to −20 mmHg of LBNP (*P* = 0.031; Fig. 3B; Main effect). There was a significant interaction effect between LBNP and trial for aldosterone. Aldosterone increased at −40 mmHg in the NFL‐HDBR trial (*P* < 0.001) and decreased at ‐40 mmHg in the Control trial (*P* = 0.028). Aldosterone was higher in the Control trial at 0 mmHg compared to the FL‐HDBR trial (*P* = 0.007) and higher in the NFL‐HDBR trial at −40 mmHg compared to the FL‐HDBR trial (*P* = 0.001; Fig. 3C). There was no effect of LBNP on vasopressin (*P* = 0.148) nor were there any differences between trials (*P* = 0.215; Fig. 3D). There was a significant increase in norepinephrine due to LBNP in all trials (*P* < 0.004, 0 vs. −40 mmHg LBNP), yet there were no differences between trials (*P* > 0.05; Fig. 3E). There was no significant effect of LBNP on atrial natriuretic peptide levels (*P* = 0.168) nor were there any differences between trials (*P* = 0.252; Fig. 3F).

## Discussion

The standard NASA fluid loading protocol did not increase plasma volume or mean arterial pressure during LBNP after either 28‐h HDBR or 4‐h sitting. However, after 28‐h HDBR fluid loading prevented reductions of resting stroke volume index and central venous pressure and prevented the elevation of both renin and angiotensin II due to HDBR. Further, fluid loading after 28‐h HDBR attenuated the production/release of renin, angiotensin II, and aldosterone due to LBNP. These changes imply that while venous return is greater after fluid loading this does not result in higher mean arterial pressure possibly due to compensatory changes in vasoconstriction or plasma hormone concentrations.

### Hemodynamics

Similar to previous studies (Hyatt and West 1977; Vernikos and Convertino 1994; Iwasaki et al. 2000; Meck et al. 2004; Waters et al. 2005; Shoemaker et al. 2012), we found that HDBR resulted in a loss of plasma volume. In contrast to Waters et al. (2005) and Hyatt and West (1977) yet in support of our hypothesis, we did not observe a restoration of plasma volume with fluid loading. Differences in trial construction and duration of HDBR (and thus magnitude of plasma volume loss) could have played a large role. However, Vernikos and Convertino (1994) also found that a fluid loading protocol similar to that used by NASA did not result in a recovery of plasma volume after 7 days of HDBR. Waters et al. (2005) did not have a control trial wherein participants underwent HDBR but did not undergo fluid loading, nor did they control for any effects of circadian rhythm. Cranston 1964 found that plasma volume was significantly higher in the afternoon, therefore the increase of plasma volume seen by Waters et al. could have been an effect of circadian rhythm. Hyatt and West (1977) performed oral fluid loading using beef bouillon which contains sodium and macronutrients (protein, fat, carbohydrates). The presence of these macronutrients could have influenced the retention of plasma volume. In the current randomized repeated measures design, we found that fluid loading after 28‐h of HDBR did not recover lost plasma volume compared to HDBR alone. The absence of plasma volume restoration could be due to the resetting of the central venous pressure operating point to a lower volume after head‐down tilt as hypothesized by Convertino et al. (2001). Indeed, HDBR alone led to a significant reduction of central venous pressure and stroke volume index (supporting previous studies of 4‐h HDBR (Edgell et al. 2012; Fischer et al. 2007)).

While the NASA fluid loading protocol increased central venous pressure and stroke volume index in the current study (suggesting increased venous return despite the loss of plasma volume), this was not great enough to have a significant effect on cardiac output index or mean arterial pressure. This increase of central venous pressure and stroke volume without a concurrent increase of plasma volume could be due to changes in venous tone and/or cardiac contractility. Importantly, Vernikos and Convertino (1994) found that oral fluid loading decreased central venous pressure and stroke volume during LBNP; however, this was after a 15‐min stand test which could have reduced central venous pressure and stroke volume due to vasodilation in active skeletal muscle.

Cowings et al. (2015) recently investigated the effects of fluid loading on the cardiovascular response to standing after 6‐h of HDBR; however, fluid loading occurred within the first hour of HDBR and standing occurred after 5 more hours of HDBR. While they did observe a transient increase of mean arterial pressure after fluid loading during HDBR (1–2.5‐h) they did not observe an effect of fluid loading on thoracic fluid volume or cardiac output at any time point. While they also observed a greater heart rate response to standing after HDBR (supporting the concept that standing and −40 mmHg of LBNP are similar orthostatic challenges), they did not observe an effect of fluid loading on any of the hemodynamic responses to standing after HDBR. Notably, the purpose of the Cowings et al. study was to investigate the optimum time at which astronauts should partake in fluid loading prior to return to Earth (i.e., orthostatic stress). Their conclusion was to perform fluid loading within 1–3‐h (Cowings et al. 2015) which corresponds with the timeline of the current study.

Our research group previously found that 4‐h of sitting without fluid loading did not change blood volume, resting hemodynamics (i.e., mean arterial pressure, heart rate, stroke volume index, cardiac output index, total peripheral resistance index), or the hemodynamic response to LBNP in men (Edgell et al. 2012); this study was conducted concurrently and in the same participants as the present study. However, in that study it was found that 4‐h of sitting without fluid loading decreased brachial vascular resistance (i.e., greater flow) whereas in the current study we did not observe a change in brachial flow in the fluid loading sitting group suggesting a peripheral vasoconstrictor effect of fluid loading (potentially due to sympathetic activation from gastric distension (Rossi et al. 1998)). Four hours of sitting without fluid loading also led to lower central venous pressure (Edgell et al. 2012) whereas in the current study 4 h of sitting with fluid loading resulted in no change in central venous pressure suggesting that fluid loading during sitting also increases central venous pressure. The proposed vasoconstriction of limb vascular beds during fluid loading could contribute to an increase of total peripheral resistance index; however, this was not observed suggesting vasodilatory changes in other unmeasured cardiovascular beds such as the brain, kidneys, or splanchnic circulation.

### Fluid‐regulating hormones

Changes in the renin‐angiotensin‐aldosterone system (RAAS) are well‐known to occur with HDBR and microgravity. After 4‐h of HDBR renin is significantly reduced (Edgell et al. 2012) in order to regulate blood volume due to the cephalic fluid shift; however, after longer periods of HDBR (7 days; (Millet et al. 2001)) or spaceflight (4 days; (Norsk et al. 1995); 57–96 days; (Hughson et al. 2016)) renin concentrations have increased. This enhanced renin response after prolonged HDBR (and enhanced angiotensin II during spaceflight (Hughson et al. 2016)) occurs with a concurrent loss of plasma volume (Norsk et al. 1995). Our data support these findings as we also found that 28‐h HDBR alone increased both renin and angiotensin II with a concurrent reduction of plasma volume, central venous pressure, and stroke volume index supporting the hypothesis of Convertino et al. (2001) that there is a lower operational set‐point for venous volume/pressure after exposure to microgravity.

Oral fluid loading after 28‐h HDBR was found to suppress resting angiotensin II and aldosterone, which would effectively inhibit the expected water and sodium retention, further supporting Convertino's hypothesis of a lower central volume set‐point after exposure to microgravity (Convertino et al. 2001). We also found a tendency (*P* = 0.079) for lower resting aldosterone after seated fluid loading compared to Control. Norsk et al. (1995) found a similar suppression of renin and aldosterone when using *i.v*. infusion of isotonic saline after ~4 days of microgravity exposure (which could have had a more robust response since there would not be the potential for fluid retention in the stomach or bladder). While our results could indeed indicate that fluid loading (in either posture) decreases aldosterone, the decrease could also be an effect of circadian rhythm since aldosterone is at its highest in the morning (Chiang et al. 1994; Hurwitz et al. 2004), as seen during the Control tests.

The increase of RAAS in response to LBNP was augmented after 28‐h of HDBR, and fluid loading attenuated the RAAS response in both the seated and HDBR positions. In contrast, Waters et al. (2005) observed a greater increase of renin with orthostatic stress after fluid loading and 12 days HDBR. Notably, the duration of HDBR between studies is considerably different. Indeed, resting levels of active renin have been shown to increase after 14 days of HDBR (Sigaudo et al. 1998) potentially augmenting its responsiveness to orthostatic stress. Since fluid loading attenuated the RAAS pathway at rest and during LBNP in the current study, one might expect a decrease of mean arterial pressure, yet this was not observed. There were no differences between trials at rest or in response to LBNP in regards to norepinephrine, atrial natriuretic peptide (ANP) or vasopressin (AVP) which supports the findings of Millet et al. (2001) who similarly saw no changes in resting ANP or AVP after 1 day of HDBR nor any changes in resting norepinephrine or its response to orthostatic stress after 7 days of HDBR. However, it is possible that enhanced peripheral vasoconstriction (or reduced vasodilation) related to an unmeasured source such as reduced nitric oxide, increased endothelin, changes in prostaglandins, and/or increased myogenic tone is preventing the expected decrease of blood pressure. Further investigations of the NASA fluid loading protocol would benefit from direct measurements of sympathetic nerve activity (especially in light of the osmopressor response to drinking water (May and Jordan 2011)) and the measurement of a greater number of vasoactive hormones.

## Conclusions

Our findings suggest that while fluid loading increased stroke volume and central venous pressure after HDBR, consistent with an increase in venous return, fluid loading did not increase resting plasma volume or mean arterial pressure. Further, fluid loading after HDBR did not prevent falls in mean arterial pressure during simulated orthostatic stress, possibly due to reduced activation of the renin‐angiotensin‐aldosterone pathway.

## Conflict of Interest

The authors have no disclosures.
